# Natural selection supports escape from concerted evolution of a recently duplicated CEACAM1 paralog in the ruminant CEA gene family

**DOI:** 10.1038/s41598-020-60425-4

**Published:** 2020-02-25

**Authors:** Jana Hänske, Tim Hammacher, Franziska Grenkowitz, Martin Mansfeld, Tung Huy Dau, Pavlo Maksimov, Christin Friedrich, Wolfgang Zimmermann, Robert Kammerer

**Affiliations:** 1 Institute of Immunology, Friedrich-Loeffler-Institute, Greifswald - Insel Riems, Germany; 2 Landesuntersuchungsanstalt für das Gesundheits- und Veterinärwesen Sachsen, Dresden, Germany; 3 Institute of Epidemiology, Friedrich-Loeffler-Institute, Greifswald - InselRiems, Germany; 4 Institute of Systems Immunology, University of Würzburg, Würzburg, Germany; 5 Tumor Immunology Laboratory, LIFE Center, Department of Urology, Ludwig-Maximilians-University, Munich, Germany

**Keywords:** Evolutionary genetics, Agricultural genetics

## Abstract

Concerted evolution is often observed in multigene families such as the CEA gene family. As a result, sequence similarity of paralogous genes is significantly higher than expected from their evolutionary distance. Gene conversion, a “copy paste” DNA repair mechanism that transfers sequences from one gene to another and homologous recombination are drivers of concerted evolution. Nevertheless, some gene family members escape concerted evolution and acquire sufficient sequence differences that orthologous genes can be assigned in descendant species. Reasons why some gene family members can escape while others are captured by concerted evolution are poorly understood. By analyzing the entire CEA gene family in cattle (*Bos taurus*) we identified a member (*CEACAM32*) that was created by gene duplication and cooption of a unique transmembrane domain exon in the most recent ancestor of ruminants. *CEACAM32* shows a unique, testis-specific expression pattern. Phylogenetic analysis indicated that *CEACAM32* is not involved in concerted evolution of CEACAM1 paralogs in ruminants. However, analysis of gene conversion events revealed that *CEACAM32* is subject to gene conversion but remarkably, these events are found in the leader exon and intron sequences but not in exons coding for the Ig-like domains. These findings suggest that natural selection hinders gene conversion affecting protein sequences of the mature protein and thereby support escape of *CEACAM32* from concerted evolution.

## Introduction

Gene families evolve by birth and death evolution^1,2^. Gene duplication provides new genetic material that can be used to test its benefit for the species without cost for changing the already existing gene^3^. The new gene may be beneficial by enhancing the gene dose or by acquiring a new function. If the enhancement of gene dosage is beneficial, the sequence of the duplicated gene has to be conserved after gene duplication. Homologous recombination and gene conversion are efficient mechanisms that favor sequence conservation of closely related sequences^4^. If sequence conservation leads to the situation, that paralogous genes are more closely related with each other than with genes of the same family in another species, even though the gene duplication preceded the speciation event, the genes are considered to evolve in a concerted way. In fact, concerted evolution is observed in many gene families, such as the CEA gene family^5^.

The CEA gene family, a member of the immunoglobulin superfamily, is one of the most rapidly evolving gene families in humans^6^. It consists of several relatively conserved genes for which orthologous genes can be assigned even in more distantly related mammals^7^ and a group of genes, which we refer to as the “*CEACAM1 paralogs*”. *CEACAM1* paralogs encode closely related extracellular IgC-like and ligand-binding IgV-like N-terminal immunoglobulin domains. Since these genes are usually more closely related within one species than with *CEACAM1* paralogs of other mammalian species it is in most cases impossible to assign orthologous genes for them in more distantly related species^8^. In some species, the *CEACAM1* paralogs are composed of two distinguished subgroups, the CEACAM and the pregnancy-specific glycoprotein (*PSG*) genes^9^. The *PSG* are specifically expressed in the placenta and were predominantly identified in mammals which have hemochorial placentae^8,10^. The domain composition of PSGs may differ between different species but their function or at least certain functions seem to be conserved^10,11^. One reason for the homogenization of *CEACAM1* paralog IgV-like domains (also called N domains) may be that the inhibitory receptor CEACAM1 is often used by various pathogens as a receptor to enter their host^12–16^ and other CEACAMs function as decoy receptors, either competing for pathogen binding or as activating/endocytic receptors on immune cells^8,17,18^.

Nevertheless, some CEACAMs seem to have successfully escaped from concerted evolution. These genes are called conserved CEACAMs and include *CEACAM16*, *CEACAM18*, *CEACAM19* and *CEACAM20*^7^. Gene conversion was not identifiable in human conserved independently evolving *CEACAM* genes^5^. Orthologs of these CEACAM genes can be found in all mammals with only a few exceptions^19^, indicating that their birth and separation from the *CEACAM1* paralogs occurred far in the past, hiding mechanisms at work during separation. Thus, the mechanisms used to escape from concerted evolution remain elusive. However, these genes may be of particular interest since some have gained important functions. For example, CEACAM16 was found to play a pivotal role in hearing, as several mutations in CEACAM16 are associated with hearing impairment^20–22^.

By characterizing the entire CEA gene family in cattle, we identified a gene, which seems to be much younger than the already known conserved CEACAMs. We suggest that this gene, further referred to as *CEACAM32* was most likely born in the most recent ancestor of ruminants. The gene is still located near the other bovine CEACAM1 paralogs in the same orientation. Nevertheless, while in phylogenetic analyses N domain exon sequences of *CEACAM32* cluster together with *CEACAM32* sequences from different ruminant species, other *CEACAM1* paralogous sequences cluster together within different ruminant species. Thus, *CEACAM32* may help to understand better, how members of a gene family can escape from concerted evolution.

## Results

### The bovine CEA gene family

**Figure 1:**
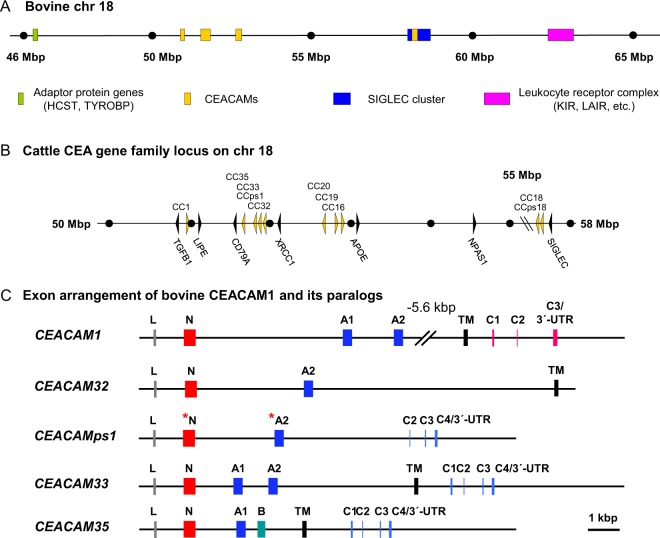
The bovine CEA gene family. (**A**) Genomic arrangement of the bovine extended Leukocyte Receptor Complex. The location and size of the genomic regions on bovine chromosome 18 containing genes encoding immune adaptor proteins, CEACAMs, SIGLEC proteins and immune receptors of the Leukocyte Receptor Complex are indicated by colored bars. **(B)** Genomic organization of the bovine CEA gene family locus. Arrowheads represent genes with their transcriptional orientation. CEACAM genes are shown in yellow, and marker genes in black. The distance indicated by dots represents 1 Mbp unless interrupted by slanted lines. **(C)** Exon arrangement of bovine CEACAM genes. The exon types are indicated by differently colored boxes. Leader sequences (L) are shown as gray, IgV-like domain exons as red (N), IgC-like domain exons as blue (A type) and green (B type) transmembrane domain exons as black boxes (TM). The exons encoding the cytoplasmic domain with an ITIM or an ITAM-like motif are shown in red and blue, respectively. The presence of deletions/insertions in exons causing reading frame shifts is indicated by an asterisk.

In order to identify bovine CEACAMs we performed similarity searches using three different bovine genomic sequence databases (*Bos taurus*, *Bos indicus* and *Bos grunniens*) using Blast and Blat algorithms as described previously^8^. By comparing data from these genomes, we predicted the coding sequence (CDS) of eight CEACAM genes and two pseudo genes. We identified orthologous genes to human *CEACAM1*, *CEACAM16*, *CEACAM18*, *CEACAM19*, *CEACAM20* and three putatively functional *CEACAM1* paralogs, *CEACAM32*, *CEACAM33*, and *CEACAM35*. The two putative pseudogenes have stop codons in multiple exons, indicating that they do not encode functional proteins. According to the current genome assembly in *Ensembl* (Cow (UMD3.1)) the chromosomal location of the CEA gene family is syntenic to the CEA gene family in other mammals located in the extended leucocyte receptor complex on chromosome 18 (Fig. 1A). *CEACAM1* is the only member of the CEA gene family located between *TGFB1* and *LIPE*. All *CEACAM1* paralogs including one pseudogene are positioned in one cluster flanked by the *CD79A* and *XRCC1* genes (Fig. 1B). The second pseudogene is a *CEACAM18* paralog located next to the *CEACAM18* gene within the *SIGLEC* cluster (Fig. 1B). The location of the orthologous genes *CEACAM16*, *CEACAM19* and *CEACAM20* is similar as in other mammals^7,8^.

The orthologous CEACAM genes *CEACAM16*, *CEACAM18*, *CEACAM19* and *CEACAM20* have the same exon arrangement as previously described in other mammals^8^ (Fig. S1). In contrast, *CEACAM1* in cattle does not contain a B domain exon (255 bp) commonly found in other mammalian *CEACAM1* genes as previously described^23^. The *CEACAM1* paralog *CEACAM32* has a unique transmembrane domain which significantly differs from the transmembrane domains found in CEA gene family members with immune receptor tyrosine-based inhibitory motifs (ITIM) or immune receptor tyrosine-based activation-like motifs (ITAM-like). CEACAM33 and CEACAM35 contain a transmembrane domain, which is very similar to that of human CEACAM3, CEACAM4, and CEACAM21. Human CEACAM3 and CEACAM4 have a cytoplasmic tail with an ITAM-like motif encoded by four exons. Similar exons could be identified in the *CEACAM33* and *CEACAM35* genes. *CEACAM35* is the only *CEACAM1* paralog which contains a B domain exon in cattle (Fig. S1). CEACAM34 described in a previous paper^8^ is no longer in the Whole-genome shotgun contigs (wgs) database at NCBI which is in accordance with our inability to amplify any fragment with CEACAM34-specific primers from cDNA of different tissues or genomic DNA from different cattle. These results suggest that *CEACAM34* does not exist as a separate gene in the cattle genome or that the sequence information previously available was incomplete. A detailed depiction of the exon arrangement of all CEACAM genes is shown in Figs. 1C and S1A.

### Differential expression of bovine *CEACAM1* paralogs

**Figure 2:**
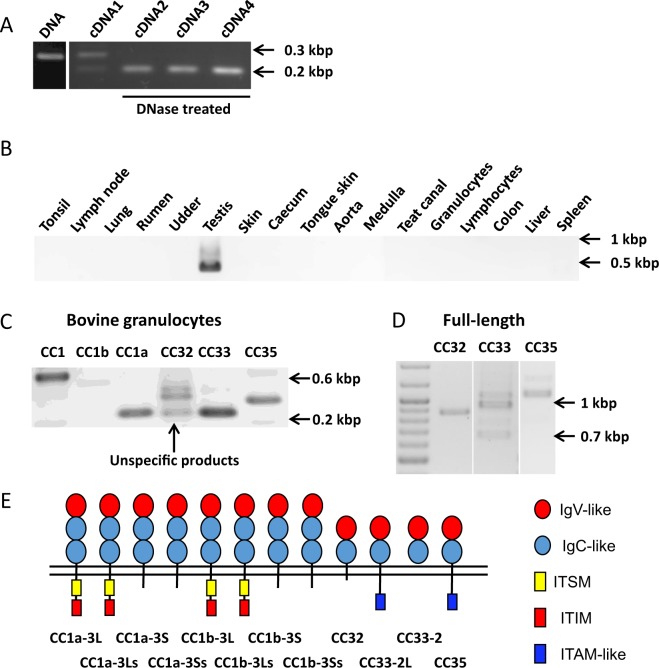
Expression and splicing of bovine CEACAM1 paralogs. (**A)** One primer pair amplifies a specific fragment of GAPDH mRNA-derived cDNA and a fragment of genomic DNA from the bovine housekeeping gene *GAPDH*. Both fragments differ in size. **(B)** Bovine CEACAM32 mRNA was specifically detected in testis by PCR. **(C)** *CEACAM33* and *CEACAM35* are expressed in granulocytes. **(D)** Amplification of full length cDNA of bovine CEACAMs. Note the different splice isoforms of CEACAM33. **(E)** The domain organization of bovine CEACAM1 and its paralogs as determined by cDNA cloning and sequencing. The signaling motifs in the cytoplasmic domains are schematically shown as blue (ITAM-like motif, no acidic amino acid present at position −1 to −3 from first Y in *bona fide* ITAM consensus motif E/Dx0-2YxxL/Ix6-8YxxL/I), red (immunoreceptor tyrosine-based inhibition motifs) and yellow boxes (immunoreceptor tyrosine-based switch motif). L and S splice variants differ by the presence or absence of a 53 bp cytoplasmic exon. Ls and Ss splice variants differ in addition by usage of alternative splice donor sites in the transmembrane domain exon^23^. CC, CEA-related cell adhesion molecule. L, long cytoplasmic variant; S, short cytoplasmic variant. Images in A, B, C and D are grouped from different gels indicated by white spaces. Gels in A, B, C, D, are cropped, ‘full-length gels are presented in Supplementary Fig. 2.

First, we designed primers for the bovine housekeeping gene *GAPDH*. The primer pair was designed to amplify a specific fragment of GAPDH mRNA-derived cDNA and a fragment of genomic DNA from the *GAPDH* gene but not from a processed pseudogene. Both fragments differ in size and therefore could be used to detect and quantify genomic DNA contamination in the cDNA preparations (Table 1, Fig. 2A).

**Table 1:** Gene-specific oligonucleotides for expression analyses and cDNA cloning of bovine CEA gene family members.

gene	oligonucleotide sequence	location of primers (exon)	size of PCR product (bp)
GAPDH	For: TGTCCACGCCATCACTGC	Exon 8	200 **(cDNA)**
	Rev: CCACAACAGACACGTTGGGA	Exon 9	286 **(genomic DNA)**
CEACAM1	For: GGGTCATCATCTCTGCACCT	Leader	510
	Rev: TGGAACCCAGTGAGTGTCAG	N domain	
CEACAM1a	For: TTCCCGAAGACGGTGCAT	Leader	246
	Rev: CCTTTGGTAGTGGCTACCCT	N domain	
CEACAM1b	For: TTCCCGAAGACGGTGCAT	Leader	251
	Rev: TGGTAGTTGCGTTAGTGTCT	N domain	
CEACAM32	For: TAACGAACGTTTCGGCTTCT	N domain	443
	Rev: TGGCGGCATACCCTACAGTT	3′UTR	
CEACAM33	For: GCAAGCAGGAGGCATGT	Leader	236
	Rev: GTGTCTACCCTATATGATGCGA	N domain	
CEACAM35	For: ACAAACTGGACATCTCTGCATAT	N domain	321
	Rev: GCTGGGCTCTTCTCTTGG	TM domain	
CEACAM32	For: TCGACCAGAAGTTCTCCTCG	5′UTR	864
	Rev: TGGCGGCATACCCTACAGTT	3′UTR	
CEACAM33	For: GTTCTCCTCACAGAGAGAGG	5′UTR	(isoform-dependent)
	Rev: GTAAGGAGACGTGTCTGGAG	3′UTR	
CEACAM35	For: TCTGGACCAGAAGTTCTCCT	5′UTR	1088
	Rev: GGGAAGTTCCTGGAGCAAA	3′UTR	**(w/o B domain)**

For: forward primer; Rev: reverse primer.

Next, we designed CEACAM-specific primers (Table 1) complementary to individual leader exon and N domain exon sequences. Using these primers we screened the expression of bovine CEACAMs in different candidate tissues, including liver, lung, skin, kidney, rumen, small intestine, large intestine, spleen, udder, tonsils, lymph nodes, granulocytes and lymphocytes. CEACAM32 mRNA was detected in testis but not in any other tissue tested (Fig. 2B). Expression of CEACAM33 and CEACAM35 was detected in bovine granulocytes isolated from peripheral blood (Fig. 2C) and at low level in other tissues (data not shown).

To substantiate our prediction of the exon composition of the CEACAM32 gene we performed 3′ RACE experiments starting from the N domain exon to identify the 3′ end of CEACAM32 cDNA. Sequencing of the cDNA obtained by 3′ RACE, revealed that the CEACAM32 mRNA encodes a unique transmembrane domain, which contains a stop codon and a poly A signal, resulting in a very short 3′ untranslated region. Based on the 3′ sequence of CEACAM32 cDNA we designed primers for the amplification of full length CEACAM32 cDNA which was then cloned and sequenced (Table 1). The CEACAM32 mRNA (GenBank accession no. MH684294) codes for a single isoform (Fig. 2D) composed of a leader sequence, an IgV-like domain (N domain), an IgC-like domain (A2 type domain), a transmembrane domain and an extremely short cytoplasmic domain consisting of four amino acids.

Amplification and sequencing of the full length CEACAM33 cDNA indicated that four different splice isoforms exists (Fig. 2D). However, we could only confirm two of these isoforms by cDNA cloning. One CEACAM33 splice variant contains the ITAM-like motif (GenBank accession no. MH684295), while another isoform (GenBank accession no. MH684296) lacks the transmembrane domain indicating that it encodes a secreted protein (Fig. 2E).

CEACAM35 (GenBank accession no. MH684297) exist as one isoform (Fig. 2D) composed of a leader exon, an N domain exon, one IgC-like domain (A1 type) exon, a transmembrane domain exon and four cytoplasmic domain exons. The four cytoplasmic domain exons code for a ITAM-like motif slightly different from the ones found in CEACAM33 cDNA. The B domain exon of the CEACAM35 gene identified as a putatively spliceable exon in the genome (Fig. 1C) was found to be not included in analyzed mRNAs. The putative proteins encoded by bovine CEACAM1 and its paralogous genes are depicted in Fig. 2E.

### Phylogenetic relationship of bovine CEACAMs

**Figure 3:**
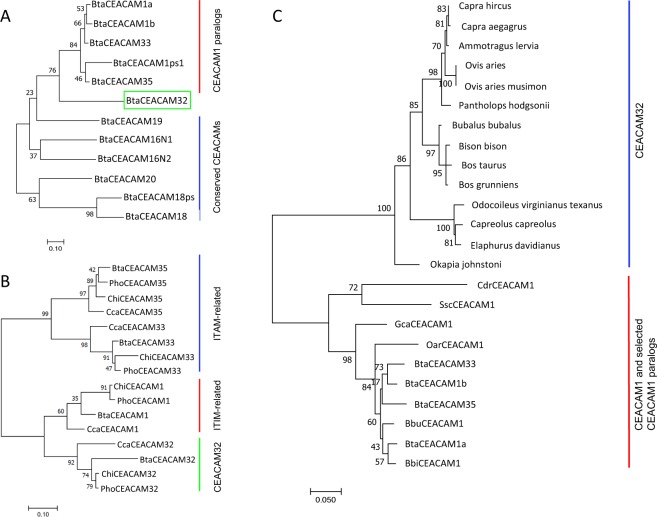
Phylogeny of bovine CEACAMs. Phylogenetic trees were constructed using the maximum likelihood (ML) method with bootstrap testing (500 replicates). **(A)** Evolutionary relationship of the Ig-V-like N domains of bovine CEA family members. Multi-alignment of N domain nucleotide sequences was performed using Muscle implemented in MEGA6/MEGA7. **(B)** Relationship of the transmembrane domain nucleotide sequence of BtaCEACAM32 with other transmembrane sequences of the CEA gene family of various species. Sequences from the following species were used: Bta (*Bos taurus*, cattle); Cca (*Capreolus capreolus*, western roe deer; Chi (*Capra hircus*, domestic goat); Pho (*Pantholops hodgsonii*, Tibetan antelope). **(C)** Relationship of the IgV-like domain nucleotide sequences derived from *CEACAM32* and other *CEACAM1* paralogs from artiodactyls. Branch lengths are proportional to the degree of inferred evolutionary change. Numbers at the nodes indicate the percentage of trees in which the associated taxa clustered together. The scale at the bottom of the tree indicates the number of substitution per site. The CEACAM32 N domain exon sequences of ruminants cluster clearly together and are separated from other CEACAM1 paralogs. Three letter code abbreviation for species: Bbi, *Bison bison*, American bison; Bbu, *Bubalus bubalis*, Asian water buffalo; Bta, *Bos taurus*, cattle; Cca, *Capreolus capreolus*, western roe deer*;* Cdr, *Camelus dromedarius*, Arabian camel; Gca, *Giraffa camelopardalis tippelskirchi*, Masai giraffe; Oar, *Ovis aries*, domestic sheep; Ssc, *Sus scrofa*, pig.

We compared the IgV-like (N domain) exon nucleotide sequences of all bovine CEACAMs (Fig. 3A). The CEACAM N domains of CEACAM1 paralogs cluster together with those of the CEACAM1a and b alleles while the N domain exon sequences of conserved CEACAMs are more distantly related. The N domain exon sequence of the inhibitory receptor CEACAM1 was most closely related to that of the activating receptor CEACAM33 followed by the second activating receptor CEACAM35. The N domain exon sequence of *CEACAM32* showed the greatest difference to that of the N domain exon of *CEACAM1* and of all CEACAM1 paralogs (Fig. 3A). We further determined the relationship of IgC-like domain exons of bovine CEACAMs (Fig. S3). CEACAM1 and CEACAM33 contain two IgC-like domains of the A1 and A2 type. CEACAM35 is composed of an A1 and a B domain. The IgC-like domains of CEACAM32 and the CEACAMps are of the A2 type (Figs. 1C; S3). Next, we analyzed the relationship of the transmembrane exon nucleotide sequences of bovine CEACAM1 paralogs with that of other species (Fig. 3B). As previously observed, two main forms of transmembrane domain exons can be discerned: one cluster is composed of TM domain exon sequences present in genes encoding ITIM motifs and the other containing the transmembrane domain exons associated with exons which encode a ITAM-like signaling motif. The transmembrane domain exon sequence of *CEACAM32* is more closely related to the *CEACAM1*-related transmembrane domain exon sequence but is clearly separated from the *CEACAM1* transmembrane exon cluster (Fig. 3B). When we compared all identified N domain exons of *CEACAM32* with N domain exons from *CEACAM1* and closely related *CEACAM1* paralogs of selected mammalian species we found that all *CEACAM32* N domain exons cluster together while N domain exons of *CEACAM1* and other bovine *CEACAM1* paralogs formed a separated cluster (Fig. 3C).

### Phylogenetic history of the bovine CEA gene family

We used the composition of mobile elements within the different bovine CEACAM genes to analyze the phylogenetic history of bovine CEACAMs (Figs. S4, S5). *CEACAM33* contains most of the elements at the same positions as in *CEACAM35* indicating that *CEACAM33* and *CEACAM35* are the result of a duplication event. Interestingly further modifications of *CEACAM33* seem to have taken place, since the B domain exon is replaced by an A2 domain exon, which is most closely related to the A2 domain exon of *CEACAM1* and/or *CEACAMps* (Fig. S3). The mobile elements around the N domain exons (i.e. in introns 1 and 2) suggest a close relationship between *CEACAM1* and *CEACAM32*. On the other hand, a similar origin of the transmembrane domain exon of these two genes is not supported by the surrounding mobile elements (Fig. S4). Based on the mobile elements found in the CEACAM1-related pseudogene it is closer related with the ITAM containing CEACAMs, *CEACAM33* and *CEACAM35* than with *CEACAM1* and *CEACAM32* (Fig. S4). Mobile elements of conserved CEACAMs indicate that the duplication of CEACAM18 was a rather recent event and that the other conserved CEACAMs evolved separately for quite a while (Fig. S5).

**Figure 4:**
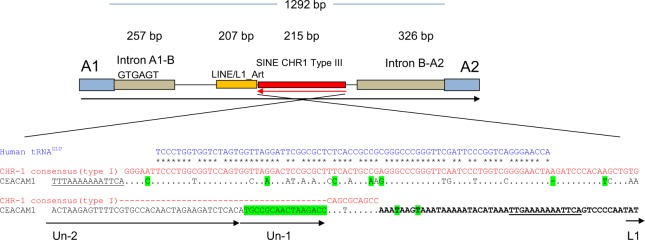
Intron between exon A1 and A2 of CEACAM1 contains retro(trans)posons. The intron between A1 and A2 starts with a sequence similar to the intron between the *CEACAM1* A1 and B exons in other species followed by a LINE/L1_Art sequence and a SINE (CHR-1 family of SINEs) and ends with a sequence that is similar to the 3′ region of the intron between the B and A2 exons. The LINE sequence is artiodactyl-specific while the SINE of the CHR-1 family is specific for Cetacea, Hippopotamidae, and Ruminantia. Similarity between SINE CHR1 (red) and human tRNA for glutamine (blue) and the insertion in bovine *CEACAM1* (black) is shown. Sequence characteristics defining SINE CHR-1 type III are indicated in green. SINE, short interspersed repetitive elements; LINE, long interspersed repetitive elements; CHR-1 family of SINEs (Cetacea, Hippopotamidae, and Ruminantia); tRNA-unrelated region composed of Un-1 and Un-2 are indicated by arrows^44^.

**Figure 5:**
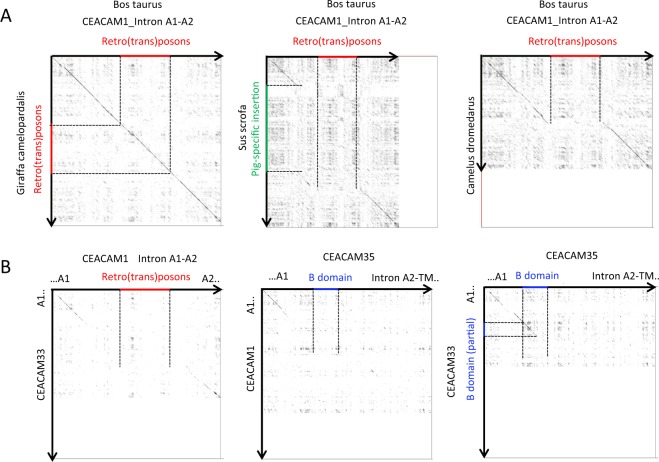
The evolutionary history of bovine *CEACAM1* paralogs. (**A)** Comparison of the A1-A2 intron sequence of *CEACAM1* from different artiodactyl species. Sequences were aligned using the *dotlet* online tool. Black diagonal lines indicate sequence similarity of longer stretches. Stippled and colored lines demarcate regions of interest. Note the absence of the retroposon sequences from the A1-A2 intron region of pig and camel *CEACAM1* and the B exon and flanking regions from cattle *CEACAM1*. (**B**) Comparison of the A1-A2 intron sequence of bovine *CEACAM1* and *CEACAM33* with the genomic region of *CEACAM35* between A1 and the transmembrane exon. Note the presence of a partial B exon sequence in *CEACAM33*.

The loss of the B domain exon in bovine *CEACAM1*, the integration of different artiodactyl-specific mobile elements (Fig. 4), and the cooption of the transmembrane domain exon of *CEACAM32* into the ruminant CEA gene family are additional genetic markers which allowed further analysis of the evolutionary history of the bovine CEA gene family. Previously we have speculated that B domain exon loss is due to the insertion of mobile elements^23^ (Fig. 4). However, when we analyzed *CEACAM1* from other artiodactyls, i.e. pig (*Sus scrofa*), alpaca (*Vicugna pacos*), and the Wild Bactrian (*Camelus ferus*) and Arabian camel (*Camelus dromedarus*), two old world camelids, we also did not find B domain exons in the *CEACAM1* genes (Fig. 5A; data not shown). Thus loss of the B domain of CEACAM1 occurred most likely in the common ancestor of all artiodactyls about 50–60 million years ago (mya). On the other hand, similar mobile elements as found in the A1-A2 intron of bovine *CEACAM1* were detected in *CEACAM1* of goats (*Capra hircus*), sheep (*Ovis aries*) and giraffe (*Giraffa Camelopardalis tippelskirchi*) but not in *CEACAM1* of pigs and camelids (Fig. 5A). This suggests that the B domain exon loss occurred before mobile elements were integrated into the A1-A2 intron of the common ancestor of ruminant *CEACAM1*.

Furthermore, in the data base *CEACAM35* has a complete B domain exon while *CEACAM33* contains only part of the B domain exon from the 5′ region (Fig. 5B). This indicates that the duplication event which created *CEACAM35* took place before the *CEACAM1*-like ancestor lost its B domain. *CEACAM33* seems to be younger than *CEACAM35* and may have been evolved by duplication of a *CEACAM35* ancestor followed by further modifications.

A close relative of the transmembrane domain exon of bovine *CEACAM32* was found in bovidae, cervidae and in some giraffidae i.e. in okapi but not in giraffe and not in suidae and camelidae. This data suggests that the birth of *CEACAM32* took place in the most recent ancestor of ruminants.

Two different *CEACAM1* alleles exist in cattle, *CEACAM1a* (GenBank accession no. AY345127) and *CEACAM1b* (GenBank accession no. AY487416). The alleles differ mainly in their N exon sequences by a number of non-synonymous mutations and most remarkably by an in frame 9-nucleotide deletion in the *CEACAM1a* allele. Interestingly, the *CEACAM1a* allele was found in the wgs database for *Bos taurus* and *Bos indicus* but not for *Bos grunniens*, *Bison bison*, and *Bubalus bubalis*. In contrast, the *CEACAM1b* allele was found in the database of all five bovine species. Taken together, this indicates that the *CEACAM1b* allele is the original bovine *CEACAM1* allele and that the *CEACAM1a* allele appeared first in the ancestor of domestic cattle 0.2–1 mya^4^ ago.

### Concerted evolution and gene conversion between CEACAM1 paralogs

**Figure 6:**
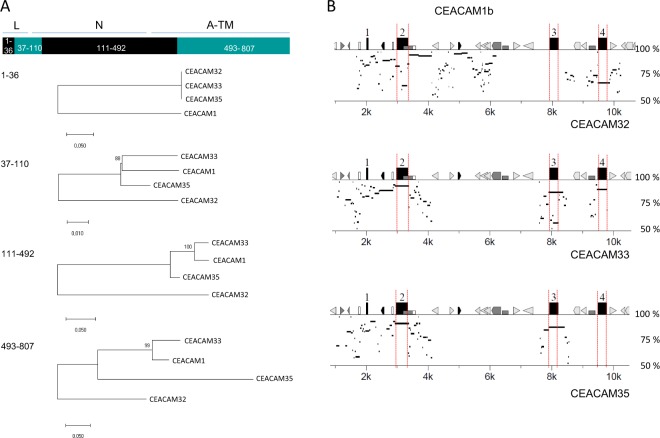
Recombination and sequence similarities of bovine *CEACAM1* paralogs. (**A)** Recombination breakpoints in the coding sequence of bovine *CEACAM1* paralogs as determined by GARD implemented in the datamonkey software. Three statistically significant breakpoints were detected schematically depicted in the graph on the top. Each region was separately analyzed for the phylogenetic relationship of *CEACAM1* and its paralogs. The region from nucleotides 111–492 coding for the main part of the N domain showed the most pronounced distance between *CEACAM32* and the other *CEACAM1*-related genes. **(B)** The nucleotide sequence of *CEACAM1* starting -2000 bp upstream of exon 1 including exons encoding the extracellular part was compared with that of the corresponding region of *CEACAM1* paralogs. For contiguous stretches of nucleotides conserved between the gene pairs using a sliding window, the degree of identity was calculated and displayed as horizontal lines. The location of *CEACAM1* exons is indicated by numbered boxes and highlighted by red lines. Note, that the sequence similarity between *CEACAM1* and *CEACAM32* is highest in intron sequences, while the similarity of *CEACAM1* sequences with that of *CEACAM33* and *CEACAM35* is highest for the exon sequences. The different repeat sequences are indicated by differently shaped forms.

**Figure 7:**
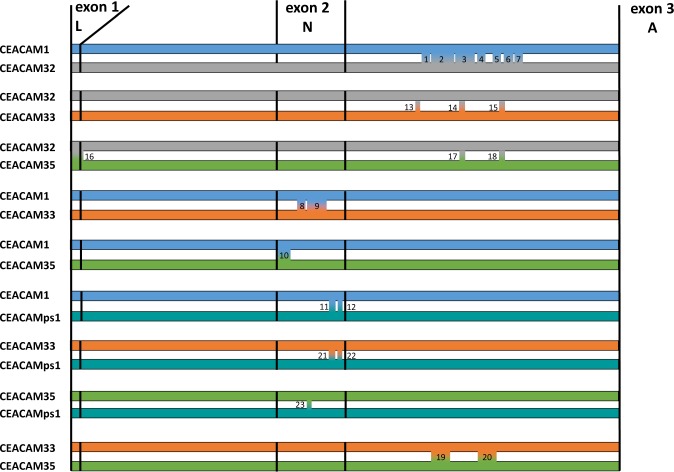
Gene conversion of bovine *CEACAM1* paralogs. Gene conversion was analyzed using GENECONV. Sequences of all bovine *CEACAM1* paralogs starting from the leader exon (exon 1) to the first ~1000 nucleotides from the intron between the N domain (exon 2) and A domain (exon 3) exon were aligned using muscle. Gene conversion was detected by GENECONV. 23 gene conversion events were detected using default parameters. Events were numbered from 1 to 23. Gene conversions between two *CEACAM* genes are depicted. The direction of gene conversion is not shown. Statistical support can be found in Table 2. Note the presence of gene conversion events in the intron sequence of *CEACAM32* and the absence of gene conversion in the N domain exon of *CEACAM32*.

The phylogenetic analysis of the N domains of bovine CEACAM1 paralogs indicates that the N domains of CEACAM1, CEACAM33 and CEACAM35 exhibit concerted evolution while the N domain of CEACAM32 evolved independently. To better understand the mechanism that allows the independent evolution of *CEACAM32* we analyzed recombination and gene conversion events that may have taken place between bovine *CEACAM1* paralogs. First, we searched the protein-coding region of bovine *CEACAM1* paralogs using GARD. Three breakpoints were identified one was in the leader sequence one in the N domain and one in the A1 domain exon. When we compared the sequences between these breakpoints, we observed that *CEACAM32* differs from *CEACAM1*, *CEACAM33* and *CEACAM35* in particular in the N and in the first IgC-like domain exons (Fig. 6A). Next, we used the PipMaker software to compare the whole sequences of the *CEACAM1* paralogous genes. As shown in Fig. 6B *CEACAM32* and *CEACAM1* N exon sequences differ strongly. However, high similarity between *CEACAM1* and *CEACAM32* was found in the intron sequences particular around the N domain exon (Fig. 6B upper panel). In contrast, sequence similarities between *CEACAM1* and *CEACAM33* and *CEACAM35* were most pronounced in the exon sequences (Fig. 6C middle and lower panels). Finally, we used GENCONV to detect putative gene conversion events between *CEACAM1*, *CEACAM32*, *CEACAM33*, *CEACAM35 and CEACAMps1*. 23 gene conversion events were detected by GENECONV in the region starting at the leader exon and ending after ~1000 nucleotides of the intron following the N domain exon (Fig. 7 and Table 2)). Gene conversion events were detected for all *CEACAM1* paralogs at the N domain exon except for *CEACAM32* (Fig. 7). Remarkably, gene conversion between *CEACAM32* and other *CEACAM1* paralogs were detected, but they were restricted to the leader exon and the intron sequence following the N domain exon (Fig. 7). We used only the region of the *CEACAM1* paralogs for the analysis of gene conversion where the sequence similarity was high enough to guarantee a high quality alignment. All gene conversion events located around the N domain exon have a high statistical support Table 2. These results demonstrate that *CEACAM32* is still involved in gene conversion and, therefore, in concerted evolution of *CEACAM1* paralogs, however, since gene conversion only affects noncoding regions and the leader exon of *CEACAM32*, the mature CEACAM32 protein has escaped concerted evolution.

**Table 2:** Gene conversions (global inner fragments) detected with GENECONV.

#	sequences	Sim P value	BC KA P value	Begin*	End*	length
1	CEACAM1; CEACAM32	0.0159	0.02575	**1843**	**1888**	46
2	CEACAM1; CEACAM32	0.0000	0.00000	**1890**	**2007**	118
3	CEACAM1; CEACAM32	0.0000	0.00000	**2009**	**2112**	104
4	CEACAM1; CEACAM32	0.0005	0.00128	**2156**	**2205**	50
5	CEACAM1; CEACAM32	0.0054	0.00948	**2259**	**2302**	44
6	CEACAM1; CEACAM32	0.0000	0.00000	**2319**	**2392**	74
7	CEACAM1; CEACAM32	0.0000	0.00011	**2507**	**2559**	53
8	CEACAM1; CEACAM33	0.0112	0.01754	1182	1236	55
9	CEACAM1; CEACAM33	0.0000	0.00000	1238	1352	115
10	CEACAM1; CEACAM35	0.0112	0.01754	1076	1149	74
11	CEACAM1; CEACAMps1	0.0042	0.00759	1302	1341	40
12	CEACAM1; CEACAMps1	0.0042	0.00759	1399	1413	15
13	CEACAM32; CEACAM33	0.0138	0.02231	**1819**	**1839**	21
14	CEACAM32; CEACAM33	0.0138	0.02231	**2041**	**2071**	31
15	CEACAM32; CEACAM33	0.0075	0.01075	**2285**	**2315**	31
16	CEACAM32; CEACAM35	0.0009	0.00223	**1**	**99**	99
17	CEACAM32; CEACAM35	0.0209	0.03649	**2041**	**2071**	31
18	CEACAM32; CEACAM35	0.0123	0.01814	**2285**	**2315**	31
19	CEACAM33; CEACAM35	0.0001	0.00032	1887	1988	102
20	CEACAM33; CEACAM35	0.0003	0.00083	2173	2322	105
21	CEACAM33; CEACAMps1	0.0081	0.01335	1302	1341	40
22	CEACAM33; CEACAMps1	0.0232	0.04000	1400	1413	14
23	CEACAM35; CEACAMps1	0.0270	0.04242	1238	1255	18

*Italic, gene conversion within the N domain exon (exon 2); bold, gene conversions with CEACAM32; Sim P value, Simulated P-values based on 10,000 permutations; BC KA P value, Bonferroni-corrected KA (BLAST-like) P-values.

### Natural selection of the putative ligand-binding domain of CEACAM32

**Figure 8:**
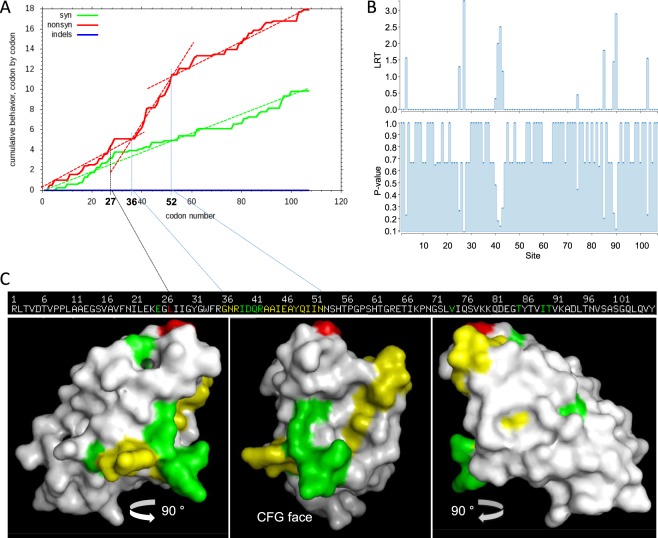
Evolution of the putative *CEACAM32* ligand-binding domain. (**A**) Codon aligned *CEACAM32* N domain exon sequences (excluding the sequence coding for the leader) from 14 ruminant species were analyzed for accumulation of synonymous (green curve) and non-synonymous substitutions (red curve). Note the rapid accumulation of nonsynonymous mutations around codon position 27 and from 36 to 52. **(B)** For the detection of individual sites under positive selection we used the “mixed effects model of evolution” software (MEME). In the upper panel results from the likelihood ratio test statistics for episodic diversification (LRT) are shown. In the lower panel the asymptotic p-value for episodic diversification are shown. **(C)** 3D modeling of bovine CEACAM32 N domain. Codon 27 which was the only found to be under episodic selection for diversification at a significance level of 0.1 using MEME is shown in red. Codons with a LRT > 0 and a p-value > 0.1 are shown in green. Codons from the region between site 36 to 52 that do not have a LRT > 0 are highlighted in yellow. The 3D image in the middle shows the CFG face which is the area of ligand interaction for most CEACAMs. Left and right images were turned 90°.

One mechanism that may avoid concerted evolution of coding sequences of the ligand-binding N-domain is natural selection as changes in intronic regions are in general better tolerated than changes in exonic regions. We used the SNAP program to calculate the ratio of the rate of synonymous nucleotide substitutions per synonymous site and nonsynonymous substitutions per nonsynonymous site (dN/dS) within the N domain exons, coding for the mature (excluding leader sequences) N domain of *CEACAM32* in different species. We used sequences from 14 different ruminant species and calculated dN/dS for all pairwise combinations which resulted in a dN/dS value of 0.65 (ranging from 0.106 *Bos taurus* vs. *Bubalus bubalis* to 2.73 *Capra aegagrus* vs. *Ammotragus lervia*) which indicates on average purifying i.e. negative selection (dN/dS <1) but positive selection may occur in certain linages. In addition, we found a stretch of codons between positions 36 and 52 where enhanced accumulation of nonsynonymous substitutions was observed (Fig. 8A). In addition, the MEME application was used to analyze the sequence alignment of *CEACAM32* sequences to search for episodic positive selection. Only one codon (codon 27) was found to be under episodic positive selection i.e. selection for diversification at a significance level of 0.1 (Fig. 8B). Additional sites were identified, which were indicated to be under positive selection by the LRT (p-value > 0.1), four of them (sites 39, 40, 41, 42) are located in the region between position 36 to 52 (Fig. 8B). Modeling the structure of the CEACAM32 N domain revealed that most the codons putatively selected for diversification are placed in or near to the CFG face (Fig. 8C). The CFG face is known to be the major ligand interaction area of CEACAM N domains. Together these data suggest that natural selection has favored differentiation of the putative ligand-binding face of CEACAM32 from other bovine CEACAMs.

## Discussion

In the present study, we performed a comprehensive analysis of the bovine CEA gene family and tried to reconstruct its evolutionary history. In contrast to the equine CEA gene family where we could not identify two of the conserved CEACAMs e.g. *CEACAM18* and *CEACAM20*^11,19^, in cattle all conserved CEACAMs i.e. *CEACAM1*, *CEACAM16*, *CEACAM18*, *CEACAM19* and *CEACAM20* could be identified in the NCBI genome database. Interestingly we also identified a CEACAM18 paralog in cattle, however this paralog is most likely a pseudogene, since several stop codons were found in the coding sequence. Thus, the gene duplication of *CEACAM18* had let to pseudogenization of one paralog, putatively due to an unfavorable consequence of the enhanced gene dosage and/or the lack of a novel function upon gene duplication^2^. In cattle, *CEACAM1* paralogs have not undergone a substantial expansion as seen in other species^8,24,25^. Only four *CEACAM1* paralogs were detected and one of them seems to be a pseudogene. Two of the expressible paralogs contain an ITAM-like signaling motif in their cytoplasmic tails. Thus, they may form paired receptors with CEACAM1 as described before in other species^17,26–28^. These paired receptors are thought to be a counter measure to the use of CEACAM1 as cellular receptors by various bacterial pathogens^8,17,27^. Interestingly, CEACAM1 paralogs with ITAM-like signaling motifs in cattle show a preferential expression in granulocytes, as does human CEACAM3, which was found to be an innate pathogen receptor, which mediates uptake and destruction of bacterial pathogens^17,27^. This is in contrast to the finding in dogs were CEACAMs with ITAM-like signaling motifs were more broadly expressed^26^. Thus, bovine CEACAM33 and CEACAM35 may also be innate immune receptors for yet unknown pathogens.

Why do two different ITAM-bearing bovine CEACAMs exist? The maintenance of two ITAM-bearing CEACAMs with a very similar expression pattern may imply that two different pathogens exist that use CEACAM1 as a receptor or that both activating CEACAMs bind to different epitopes of the same pathogen. In addition, the presence of two activating CEACAMs may allow the development of more than one inhibitory receptor without losing the “protection” by the activating receptor^8,17,27^. Indeed, we had previously identified two different CEACAM1 alleles in cattle. We assumed that this is due to usurpation of bovine CEACAM1 by a virus as its cellular receptor. However, such a virus has not yet been identified^23,29^. Here we show, that the *CEACAM1a* and *CEACAM1b* alleles are found in certain *Bos taurus* and *Bos indicus* breeds, but *CEACAM1a* is absent from the genomes of *Bos grunniens*, *Bison bison*, and *Bubalus bubalis*. This finding strongly indicates that the three amino acid deletion in the *CEACAM1a* allele occurred in the common ancestor of *Bos taurus* and *Bos indicus* after separation from the ancestor of *Bos grunniens* and *Bison bison* about 150–400 kya^30^. At that time, both ITAM-containing CEACAMs already existed, since their descendants could be found in the genome of both *Bos grunniens* and *Bison bison*. Thus it may be speculated that the presence of two ITAM-bearing receptors with different ligand-binding domains favor the evolution of distinct *CEACAM1* alleles.

From an evolutionary point of view, it is remarkable that *CEACAM35* is the only *CEACAM1* paralog in cattle which contains a functional B domain exon. Since the B domain exon of *CEACAM1* is replaced by a LINE/L1_Art and a CHR1 family SINE interspersed repeat element, duplication of *CEACAM1* leading to *CEACAM35* may have occurred before this event. Thus, CEACAM1 and CEACAM35 seem to be the original paired receptors of the CEA gene family in artiodactyls. In contrast, *CEACAM33* does not have a B domain exon indicating, that *CEACAM33* either evolved by duplication of *CEACAM1* after the loss of the B domain exon or by duplication of *CEACAM35* followed by an independent loss of the B domain exon. The later possibility is supported since *CEACAM33* contains a region that exhibits sequence similarity to the sequence comprising the end of the intron between the A1 and B domain exon and the first part of the B domain exon of *CEACAM35*. In addition, the presence of similar mobile elements in *CEACAM35* and *CEACAM33* suggests that the *CEACAM35* ancestor gave rise to *CEACAM33*. However, the presence of an A2 domain in CEACAM33 implies the presence of an A2 exon in the *CEACAM35* ancestor or requires further modifications of an A2 exon-less *CEACAM33* ancestor after gene duplication possibly by recombination or gene conversion.

There is one member of the bovine CEA gene family which is of particular interest since it has a unique transmembrane domain which was not found previously in other species. Further searches in the NCBI database suggest that CEACAM32 is specific for ruminants. Thus CEACAM32 is an example of a new CEACAM born in the most recent ancestor of all ruminants about 35–50 mya^31^. Although the origin of the transmembrane domain of CEACAM32 is unknown, the novelty of this domain within the CEA gene family indicates that *CEACAM32* is the result of a gene duplication and/or exon shuffling event together with the cooption of a novel transmembrane exon. This novel gene encoding a transmembrane anchored cell surface glycoprotein has gained a unique expression pattern within the bovine CEA gene family. An exclusive expression in testis was previously also observed for *CEACAM17*, which is a muroid-specific CEACAM^7^. Taken into consideration that CEACAMs could interact with a variety of ligands we speculate that the expression in testis provides a new very specific environment for these testis-specific CEACAMs with a unique repertoire of putative ligands that may favor adaptation of the putative ligand binding domain of CEACAM to bind to a testis-specific ligand. Indeed, the relaxed or even positive selection at the CFG face fits well to the view that the ligand binding face did undergo affinity maturation to this putative new ligand or that adaptation to species-specific ligands took place. Nevertheless, we want to point out that it is well known that transcription is very permissive in testis and, therefore, duplicated genes are often transcribed in this organ^32,33^. Thus, further analysis of the role of CEACAM32 is needed to support the view that CEACAM32 plays a role in testis.

Comparison of the N domains of bovine CEACAM1 paralogs showed that *CEACAM1* and the two ITAM-like signaling motif containing CEACAMs (*CEACAM33* and *CEACAM35*) underwent concerted evolution. A similar finding was recently reported for human *CEACAM1* paralogs^5^. Furthermore, concerted evolution of the protein coding sequence of *CEACAM1*, *CEACAM33* and *CEACAM35* seems to be due to gene conversion events that affect the exon sequences and only to a minor extend the intron sequences. In contrast gene conversion events of *CEACAM32* affecting non-protein coding intron sequences are preferentially maintained during evolution. This indicates that natural selection favors maintenance of the protein sequence of the extracellular part of CEACAM1 and the ITAM-containing CEACAMs while it prevents homogenization of the extracellular part of CEACAM32 and CEACAM1. Gene conversion is supported by both small distance of genes and sequence similarity. Thus, gene conversion at certain places of the intron sequence between *CEACAM1* and *CEACAM32* may be preferred compared to *CEACAM33*, *CEACAM35* or *CEACAMps1*. On the other hand, we have noted inversions of the non-CEACAM gene locus present between *CEACAM1* and members with ITAM-like motif-encoding exons in cattle and mouse when compared with the human CEACAM gene locus^8^. This intrachromosomal inversion can be explained by a recombination event between e.g. *CEACAM1* and a ITAM-like motif-encoding CEACAM1-like gene with an transcriptionally inverse orientation (which indeed all CEACAM1-like genes exhibit; see Fig. 1B) possibly using a lopping-out mechanism comprising the non-CEACAM gene region. This mechanism could engage even genes with large physical distances, suggesting that the different distance between CEACAM1 and the individual CEACAM1 paralog is probably of minor importance for the frequency of gene conversion events.

It is of particular importance that concerted evolution within bovine *CEACAM1* paralogs is only relevant for the extracellular, ligand-binding part. Due to the contrary signaling capacity of bovine CEACAM1 and its paralogs, functional diversification of the duplicated genes already had taken place^1^. Thus their transmembrane and cytoplasmic parts are not under concerted evolution due to prominent nucleotide sequence differences. We hypothesize that the ligand-binding domain of inhibitory CEACAM1 and activating CEACAMs evolve in a concerted way in order to maintain the counter measure function of activating CEACAMs against the use of the inhibitory CEACAMs by pathogens. Since the evolution of these CEACAMs is most likely driven by pathogens which evolve very rapidly the ligand binding domains also evolve rather fast. To keep the ligand (probably pathogen adhesins) binding domains similar, these paired receptors evolve by concerted evolution mediated by gene conversion and homologous recombination. On the other hand, duplicated genes that have gained a novel expression pattern face a novel environment and may interact with novel putative ligands. Once they interact to a certain extend with these ligands further optimization by natural selection may exclude concerted evolution with the original parental gene^34^. According to the Red-Queen-Hypothesis^35^ this means not moving in concert with its paralogs means separation from them without prominent selection for diversification, as it was observed in the current investigation for CEACAM32.

Taking together analysis of the CEA gene family of cattle and other artiodactyls, provided evidence, that members of the CEA gene family can escape from concerted evolution by excluding protein coding regions of the gene from gene conversion most likely through natural selection. Thus, conserved CEA gene family members are expected to have ligands distinct from their founder gene *CEACAM1* ligands.

## Materials and Methods

### Datasets and nomenclature of genes

Sequence similarity searches were performed using the NCBI BLAST tools blastn http://blast.ncbi.nlm.nih.gov/Blast.cgi and Ensembl BLAST/BLAT search programs http://www.ensembl.org/Multi/Tools/Blast?db=core using default parameters. For identification of bovine CEACAM exons, exon and cDNA sequences from known CEACAM and PSG genes were used to search whole-genome shotgun contigs (wgs) databases limited to organisms *Bovidae*. Hits were considered to be significant if the E-value was <e-10 and the query cover was >50%. Once a wgs contig was identified that contained CEACAM-related sequences we confirmed manually the presence of the complete exon by the number of nucleotides and identification of CEACAM-typical splice site sequences. Only sequences which were considered to be complete exons were used for further analyses. In a second step we used the identified exon sequences to search the database again in order to identify all existing paralogous CEACAM genes. Once we had identified individual exons we predicted the gene structure based on the organization of known CEACAM genes. The location of different exons on the same contig was a prerequisite for considering that these exons belong to the same gene. Gene predictions were further supported by the identification of expressed sequence tags (est) and/or predictions in genome builds at NCBI and Ensemble, if available. Short exons, like exons coding for cytoplasmic tails, were identified by alignments of downstream sequences of identified transmembrane exons with cytoplasmic exon sequences of human CEACAMs. Sequence alignments for exon identification were performed using clustalw (http://www.genome.jp/tools/clustalw/). For the identification of CEACAMs from other artiodactyls the following wgs data sets were used: *Bos taurus* DAAA02 (Genome Coverage (GC): 9×; Sequencing Technology (ST): Sanger); *Bos indicus* PRDE01 (GC: 100×; ST: 454; IonTorrent; Illumina NextSeq; Illumina MiSeq), AGFL01 (GC: 52×; ST: SOLiD); *Bos mututs* AGSK01 (GC: 130×; ST: Illumina HiSeq; Illumina GA); *Bison bison bison* JPYT01 (GC: 60×; ST: 454; Illumina HiSeq); *Bubalus bubalis* AWWX01 (GC: 70×; ST: Illumina GAIIx; Illumina HiSeq; 454); *Giraffa tippelskirchi* LVKQ01 (GC: 37×; ST: Illumina HiSeq); *Okapia johnstoni* LVCL01 (GC: 30×; ST: Illumina HiSeq); *Sus scrofa* LUXX01 (95.5×; ST: Illumina HiSeq); *Camelus ferus* AGVR01 (GC: 30×; ST: Illumina GAIIx; 454 GS-FLX Titanium; SOLid 3); *Camelus dromedaries* JWIN01 (GC: 65×; ST: Illumina HiSeq); *Vicugna pacos* ABRR02 (GC: 22×; ST: Roche 454; ABI 3730); *Capra aegagrus* CBYH01; *Capra hircus* AJPT02 (GC: 175×; ST: Illumina); *Ammotragus lervia* NIVO01 (GC: 124×; ST: Illumina); *Ovis aries* AMGL02 (GC: 166×; ST: Illumina GAII; 454; PacBio RSII); *Ovis aries musimon* CBYI01 (GC: not available; ST: not available); *Pantholops hodgsonii* AGTT01 (GC: 67×; ST: Illumina); *Odocoileus virginianus texanus* MLBE01 (GC: 150×; ST: Illumina); *Capreolus capreolus* CCMK01 (GC: not available; ST: not available); *Elaphurus davidianus* JRFZ01 (GC: 114×; ST: Illumina HiSeq 2000). The CEA gene family in cattle is not well annotated; therefore, we adopted the nomenclature according to the one previously used for the CEA gene family of other mammals i.e. bovine CEACAM1 paralogs were numbered CEACAM32-CEACAM35 following the canine CEACAM numbers^8^. Gene names and corresponding sequences are summarized in (Additional File 1). The following databases were used for gene loci analyses: UMD3.1 assembly and Btau_5.0.1.

### Cells and tissues

Bovine peripheral blood lymphocytes and granulocytes were isolated from blood of healthy cattle by centrifugation through a Ficoll-Paque gradient 1.077 g/l (GE Healthcare, Chalfont St Giles, UK). Lymphocytes were taken from the interphase of the gradient and separated from monocytes by plastic adherence for one hour. Granulocytes were collected from the top of the red blood cell (RBC) pellet and further purified by RBC lysis with ammonium chloride. Different bovine tissue samples were collected from freshly slaughtered healthy cattle and stored in the RNA stabilization reagent RNAlater® (Invitrogen, Carlsbad, US) at 4 °C for 24 h or at −80 °C for long term storage.

### Reverse transcription-polymerase chain reaction analysis

Total RNA was isolated with the RNeasy® Mini Kit (Qiagen, Langen, Germany) or using the TRIzol® reagent (Life Technologies, Karlsruhe, Germany). One µg of total RNA was used for cDNA syntheses by reverse transcription (RT) using the Reverse Transcription System® (Promega, Mannheim, Germany). The RT product was amplified by polymerase chain reaction (PCR) with DreamTaq polymerase (Thermo Fisher Scientific Inc., Waltham, USA) and gene-specific primers (Metabion, Planegg-Martinsried, Germany) using standard conditions. Primers used are summarized in Table 1. Eight µl of each PCR were analyzed by electrophoresis on a 1.8% agarose gel and visualized by ethidium bromide staining.

### Bovine CEACAM cDNA cloning and sequencing

Primers used for amplification of full length cDNAs are shown in Table 1. For cDNA cloning the RT product was amplified by PCR with Easy-A High-Fidelity PCR Cloning Enzyme (Agilent) and analyzed by agarose gel electrophoresis. Specific bands were extracted from the agarose gel using QIAEX II Gel Extraction Kit (Qiagen). The PCR products were cloned using the StrataClone PCR Cloning Kit (Agilent). Plasmid DNA isolated from various clones were analyzed by PCR and sequencing. Nucleotide sequencing was performed with the BigDye Terminator Cycle Sequencing Kit (PE Applied Biosystems, Weiterstadt, Germany).

### Rapid amplification of cDNA ends (RACE)

For the amplification of the 3′ end of CEACAM32 mRNA we used the 3´ RACE System for Rapid Amplification of cDNA Ends from Invitrogen according to the standard protocol. The amplicons were isolated from an agarose gel using the QIAquick Gel Extraction Kit from Qiagen and sequenced using the BigDye Terminator Cycle Sequencing Kit (PE Applied Biosystems). This sequence was used to design specific primers to amplify full-length CEACAM32 cDNA. The sequences of the PCR products were determined by direct nucleotide sequencing.

### Phylogenetic analysis and bioinformatics

Phylogenetic analyses based on nucleotide and amino acid sequences were conducted using MEGA6 or MEGA7^36,37^. Sequence alignments were performed using Muscle implemented in MEGA7. Phylogenetic trees were constructed using the maximum likelihood (ML) method with bootstrap testing (500 replicates) and the Tamura-Nei substitution model. For comparing sequences by the diagonal plot method we used Dotlet (https://myhits.isb-sib.ch/cgi-bin/dotlet)^38^. The program PipMaker (http://bio.cse.psu.edu/) was used to identify conserved contiguous stretches of nucleotides between gene pairs and to calculate the degree of identity which is summarized as a ‘percent identity plot’^39^. For identification of mobile DNA elements we used RepeatMasker (http://www.repeatmasker.org/). Recombination between bovine *CEACAM1* and its paralogs was detected using the genetic algorithm for recombination detection (GARD) software^40^. Gene conversion was analyzed using the GENECONV program (version 1.81a)^41^. In order to determine the selective pressure on the maintenance of the nucleotide sequences, the number of nonsynonymous nucleotide substitution per nonsynonymous site (dN) and the number of synonymous substitutions per synonymous site (dS) were determined for N domain exons. The dN/dS ratios as well as the cumulative synonymous and nonsynonymous substitutions along coding regions of N domain exons from orthologous genes were calculated after manual editing of sequence gaps or insertions guided by the amino acid sequences applying the SNAP program (Synonymous Nonsynonymous Analysis Program; http://www.hiv.lanl.gov/content/sequence/SNAP/SNAP.html), which uses the modified Nei-Gojobori model with Jukes-Cantor correction. We verified the results using the JCoDa software (http://www.tcnj.edu/nayaklab/jcoda). For the detection of individual sites under positive selection we used the mixed effects model of evolution software (MEME)^42^. 3D modeling was performed with geno3D-release 2 homology modeling software^43^ and visualized by the PyMOL software (Schrödinger Inc., New York, US). The N domain of CEACAM32 was modeled using pdb2qsqA-0 and pdp116zA-0 as templates.

### Ethics approval and consent to participate

Healthy cattle were slaughtered for meat production at the abattoir “LandWert Hof Sundhagen”, not as part of this study, however we got permission from the abattoir to use the tissues for the present study. Further tissue collection was approved by the animal use committee of local authorities (Landesamt für Landwirtschaft, Lebensmittelsicherheit und Fischerei (LALLF) Rostock, Germany; 7221.3-2.1-011/13). All experiments were performed in accordance with relevant guidelines and regulations.

## Supplementary information

Supplementary Information.

## Data Availability

Nucleotide sequences from bovine CEACAMs are available at NCBI GenBank accession numbers MH684294 - MH684297.
