# Supplementary material for: Natural selection supports escape from concerted evolution of a recently duplicated CEACAM1 paralog in the ruminant CEA gene family
Source: Sci Rep. 2020 Feb 25;10:3404. doi: 10.1038/s41598-020-60425-4 (PMC7042247; doi:10.1038/s41598-020-60425-4)
Supplement: Supplementary file 1 — Supplementary Information. [file 41598_2020_60425_MOESM1_ESM.pdf]

Natural selection supports escape from concerted evolution of a recently duplicated CEACAM1 paralog in the ruminant CEA gene family

Jana Hänske[1,2], Tim Hammacher[1], Franziska Grenkowitz[1], Martin Mansfeld[1], Tung Huy Dau[1], Pavlo Maksimov[3], Christin Friedrich[1,4], Wolfgang Zimmermann[5], Robert Kammerer[1]*

[1]Institute of Immunology, Friedrich Loeffler Institute, Greifswald-Insel Riems, Germany

[2]Landesuntersuchungsanstalt für das Gesundheits- und Veterinärwesen Sachsen

Dresden, Germany

[3]Institute of Epidemiology, Friedrich-Loeffler-Institute, Greifswald-Insel Riems, Germany

[4]Institut für Systemimmunologie, Universität Würzburg, Germany

[5]Tumor Immunology Laboratory, LIFE Center, Department of Urology, University Hospital of Munich, Munich, Germany

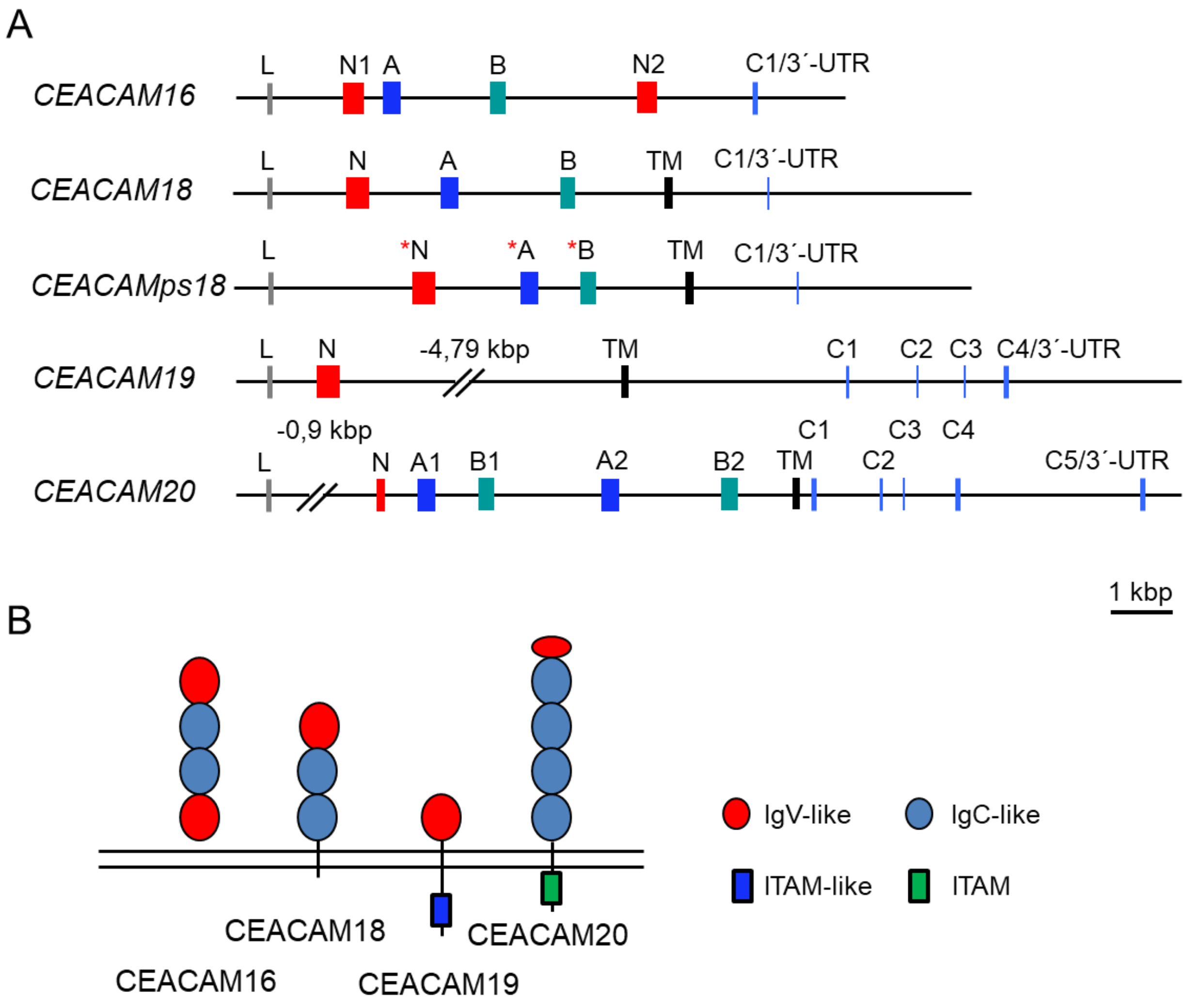

Supplementary Figure 1 - Conserved bovine CEA gene family members. (A) Exon arrangement of conserved bovine CEACAM genes. The exon types are indicated by differently colored boxes. Leader sequences are shown as gray, IgV-like domain exons as red, IgC-like domain exons as blue (A type) and green (B type) transmembrane domain exons as black boxes. The exons encoding the cytoplasmic domain with an ITAM-like or an ITAM motif are shown in blue and green, respectively. The presence of deletions/insertions in exons causing reading frame shifts is indicated by an asterisk. **(B)** The domain organization of bovine conserved CEACAMs. The signaling motifs in the cytoplasmic domains are schematically shown as green (immunoreceptor tyrosine-based activation motifs; ITAM), blue (ITAM-like motif, no acidic amino acid present at position -1 to -3 from first Y in consensus motif E/Dx0-2YxxL/Ix6-8YxxL/I).

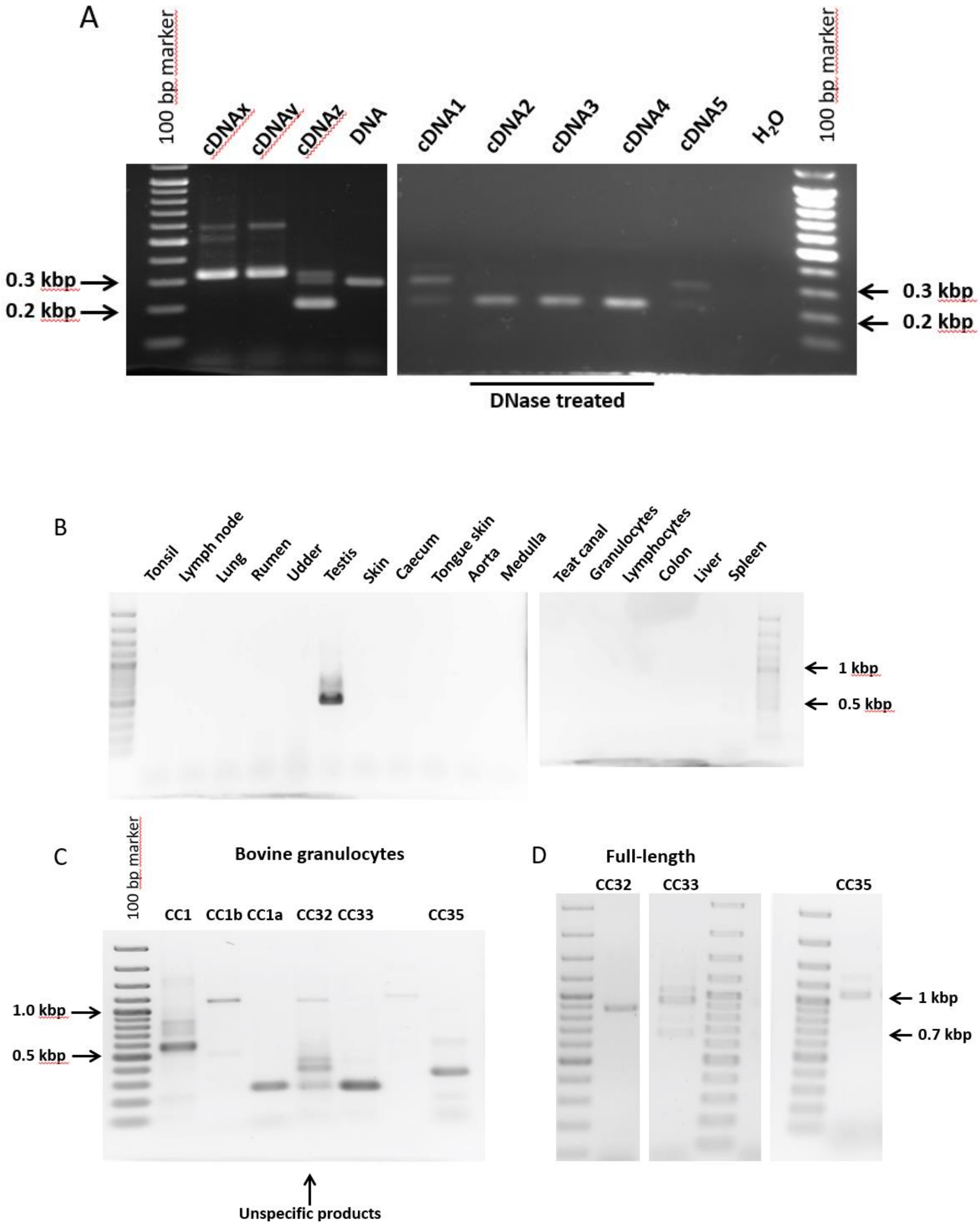

Supplementary Figure 2 - 'Full-length' gels used in Figure 1 A, B, C, D. These uncropped images are labeled as in the main text.

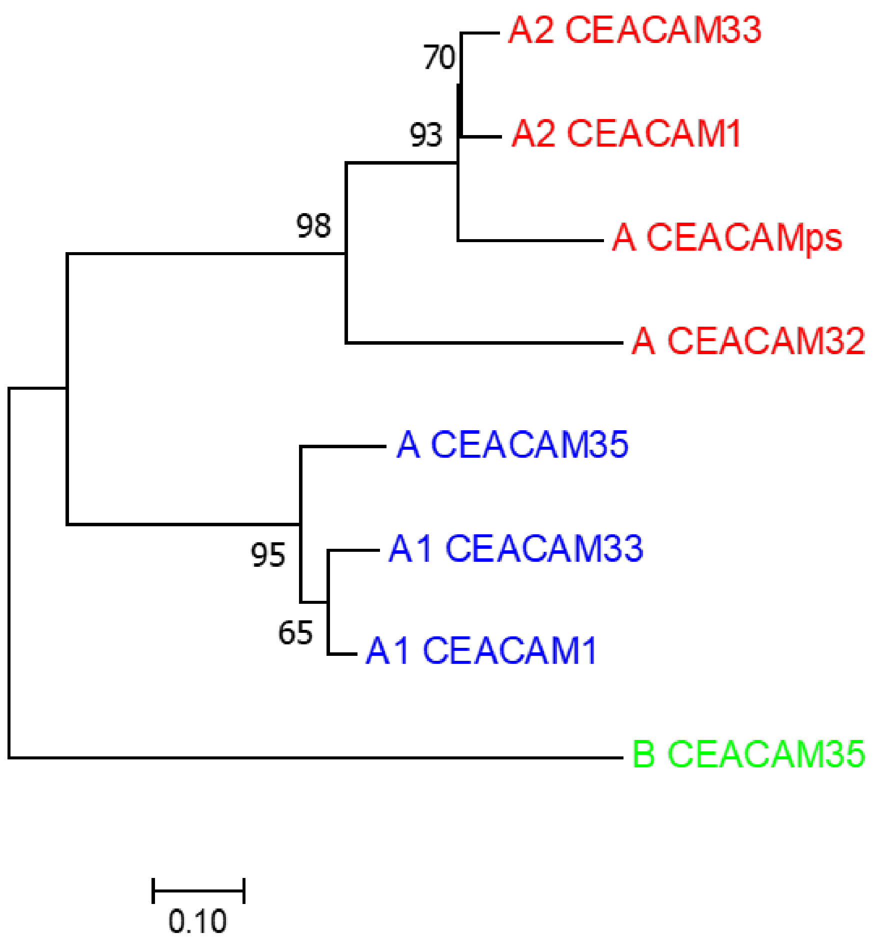

Supplementary Figure 3 – Phylogenetic relationship of IgC-like domains of CEACAM1 paralogs. The Phylogenetic tree was constructed using nucleotide sequences and the maximum likelihood (ML) method with bootstrap testing (500 replicates). Numbers at the nodes indicate the percentage of trees in which the associated taxa clustered together.

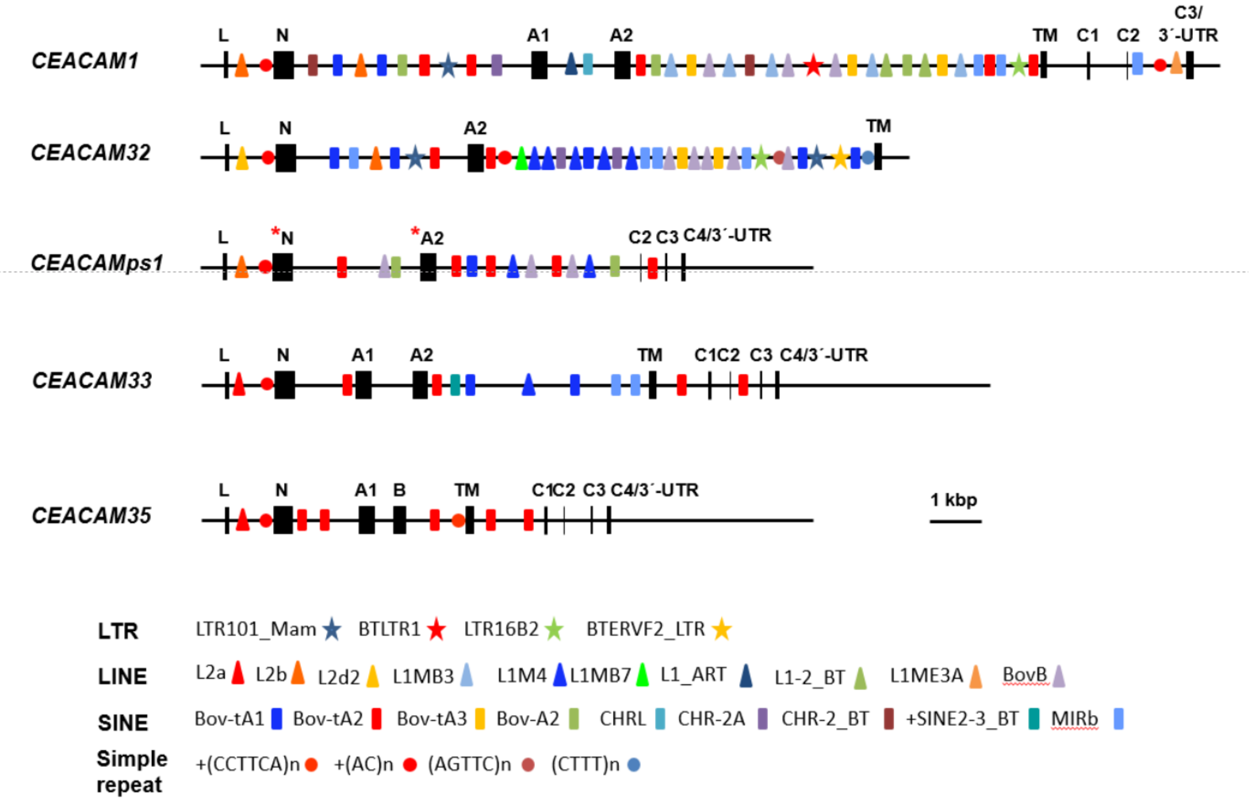

Supplementary Figure 4 – Mobile elements within bovine CEACAM1-related CEACAMs. Mobile elements and low complexity DNA sequences within bovine CEACAM1-related genes were identified using the RepeatMasker software. LTR (stars), Lines (triangles), Sines (rectangles) and simple sequences (discs) were placed in the corresponding order into introns.

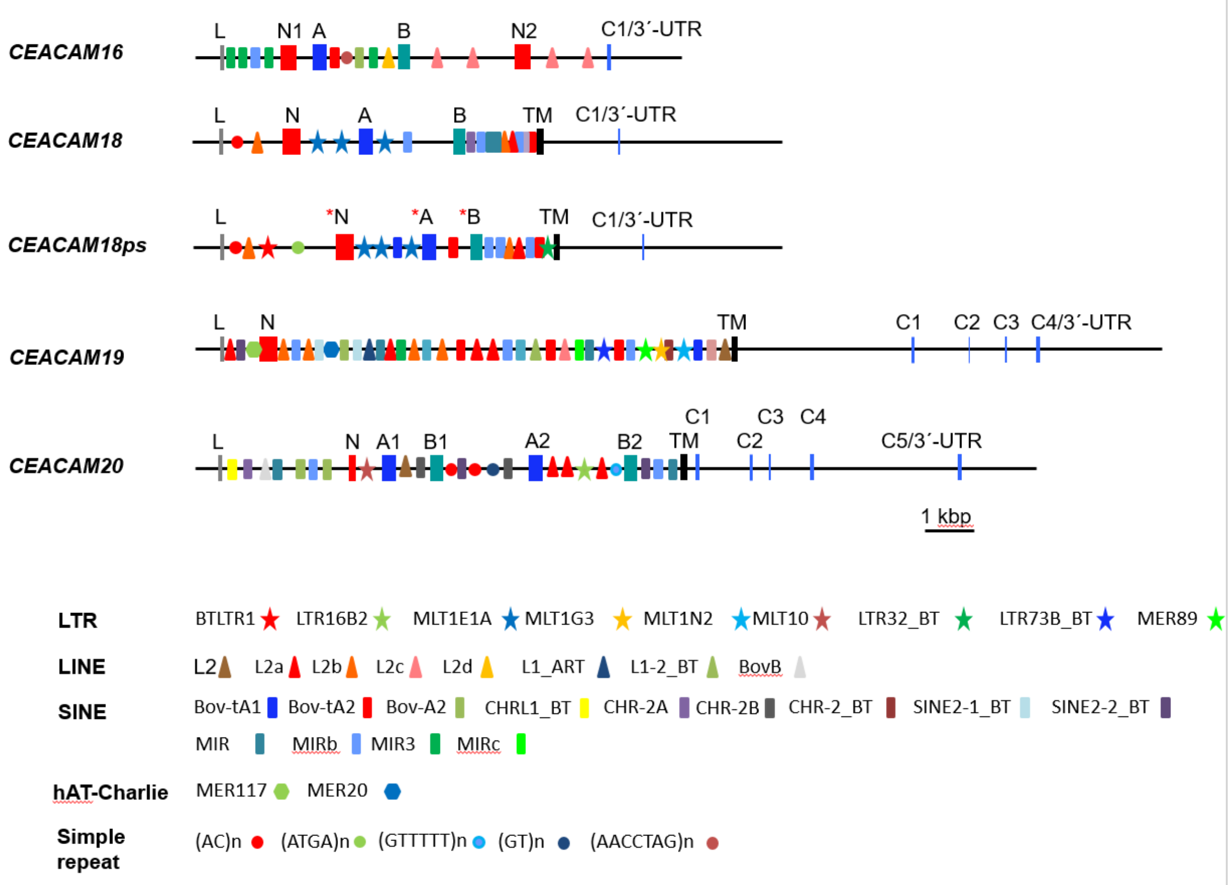

Supplementary Figure 5 – Mobile elements within bovine conserved *CEACAM* genes. Mobile elements and low complexity DNA sequences within bovine *CEACAM* genes were identified using the RepeatMasker software. LTR (stars), Lines (triangles), Sines (rectangles) and simple sequences (discs) were placed in the corresponding order into introns.

Supplementary File 1 – predicted coding sequences of bovine conserved CEACAMs

(Exon sequences are indicated by different colors)

>CEACAM16 [Bos taurus]

ATGGTCTCAGGGAAGGGAGGGGAGCCTGGGGAGGGAAGATCCGCGATCCTGAGTGCGGGGGCCGAGATCATTATCACC
CCCGAGCCTGCCCAGCCAGCCGAGGGCGACAACGTCACTCTGGCTGTCCAAGGGCTTTCGGGGGAACTGCTGGCCTAC
AACTGGTATGCGGGGCCCACGCTCAGCCTGTCTTACCTGGTAGCCAGCTACATTGTAAGCACAGGCGATGAGACCCCT
GGCCCAGCCCACACGGGGCGGGAGGCTGTGCGCCCCGACGGTGGCCTGGACATCCAGGGTGCCCTGCCTGGGCACTCG
GGCACCTACATTCTGCAGACTCTCAACAGGCAATTTCAGACGGAGGTGGGCTACGGACACATGCAGGTCTATGAGATC
CTGGCCCAGCCCGTGGTCGTGGCCAACAGCACGGCCTTGGTGGAGCGCCGGGACACCCTACACCTGACGTGCAGCAGC
CCCAGCCCTGCTGAGGTCCGATGGTTCTTCAACGGCGACGCCTTGCCCATCGCCGTGCGCCTTGGCCTGTCCCCCAAC
GGCCGGGTGCTGACCCGGCATGGCATCCGCAGGGAGGAGGCCGGCGCCTACCAGTGTGAGGTCTGGAACCCTGTCAGT
GTCAGCCGCAGCGAGCCCGTCAACCTGACCGTGTACTTTGGCCCAGAACGCGTGGCCATACTCCAGGACTCCACGACC
CGCACAGGCTGCACCATCAAAGTCGACTTCAACACGTCCCTCACGCTGTGGTGTGTGTCCCGGTCCTGCCCGGAGCCC
GAGTACGTGTGGACCTTCAATGGGCGGGCCTTAAAGAGCGACCAAGACCACCTGAACATCAGTAGCATGACAGCCACC
CAGGAGGGCACGTACACGTGTATTGCTAAGAACCCCAAGACCCTGCTCTCTGGATCTGCCTCAGTGGTGGTCAAACTC
TCTGCGGCCACGGTCATCATGACCATCGTTCCGGTGCCCACCCGGCCAATGGAGGGCCAGGACGTGACACTGACCGTC

CAGGGCTACCCCAAGGATCTGCTGGTCTATGCCTGGTACCGTGGGCCTGCCTCCGAGCCCAACCGGCTTCTCAGCCAA
CTTCCTTCCGGGAACTGGATCGCAGGCCCTGCGCACACAGGCCGGGAGGTGGGCTTCCCCAACTGCTCACTGCTGGTG
CAGAAGCTGAACCTCACAGATGCCGGCCGCTACACCCTCAAGACCGTCACGCTGCAGGGCAAGACAGAGACACTGGAA
GTGGAGCTGCAGGTGGCCCTCCTGGAGTAG

>CEACAM18ps[Bos taurus]

ATGGACCTTTCTAGACCCAGGTGCAGACTCTGGAGAAAACTAGTCCTTGTGGCCAGTCTGCTGGCCTGTGAGATCACC
CAGGCCTCCAGCCAAATGTACATCAGCCCAGACCCATTCACAGGTTTGAAGGGATACCGGAGCCTCCTGATTTTCAAC
AATGCCCATAAAAATGTTCAGGAGTATAGTTGGCACCAGGGTACAAATGACACTGAGGAAAATCTTATTATCAGCTAC
AACACCACATCTCATTCCCAGCAGAATGGGCCTAGGTACAGTGGCCGGGAAAGAGTGTCCCCTACAGGTGCCCTGCTG
ATCAAGGAGTCTTGATTAAATGATACAGGGAACTACACCGTGCGGGTGGACGCTATCAATGACACCCAGAGAGCAAAT
GTCTGGCTCAAGATTCGAGTGTTTGAAATCCCAGGCATCTCAGTCAACACCAGCTACGCAGGACTGAATGTAGACTCC
GTGGCTGCCATCTGCCAGACGAACGACACGAATGTCTACTGGTTTGTAGGCTACACACAGGTGTCCAGCTCTGAACGC
GTGACCATCTCTCCAGACACTAAGACCCTCATCATCAAAAGGATCAGGAGCAACGACTCACTGCTGCAGTGCTCAGGG
TCTCCCAGGGACATCATGACAAAACAGGCAGAGAGTGTGGCATGAGACACCGGCATCCTGCCTGCTGAGGTCAGCTCC
CAAGTGAAGATGGAGTATCTTTCTGATGGCAGACCGCAAATCAAGTACCACTGGATCCACAATATCTCTGTCCTGAGC
TTCTCAGAGAAGAACATCACCCTCCTAAGTCTGACCTGGGACCAGATGGGCAGATACAGGTGCATCCCGGAGAACTCT
GCCACCCACGAGATCTTGTACGATGAAGTCCAGGTCCAGGCCCACATGCCCAAGATTAGCCAAGATGTCACCATGTCA
ACACTGCTCCTGAACTTCCTCATGACGTGGTCAATCTTGGGCTATGTCTTCCTCTTTACACTCATCCTTCTGGGCCTC
ATCAGATGTTATTCCACCAGTAAATAGGATTCCAACTTCTCCACTTCCTTAACCTGACCCTGTGGGACCATCAGAGAG
GCTGCAAATATGCCCCATCTCAGCATCGCCAGTAA

>CEACAM18 [Bos taurus]

ATGGACCTTTCCAGACCCAGGTGCAGACTCTGGAGGGAACTGGTCCTTGTGGCCAGTCTGCTGGCCTGTGGGATCCGC
CAGGCCTCCAGCCAAATCTACATCACCCCGGATTCACTCATCGGAGTGGAAAGATATTCGAGCTCACTGGCCATCGAG
AACGCCCCTGAAGATGTTCAGGAATACAGCTGGCACCGAGGTGCAAATGACACTGAGGAAAATCTGATTATCAGCTAC
AACGCCACATCTCATTCCAGGCGGGATGGGCCCATGTACAGCGGCCGGGAAAGTGTGTCCATTAGAGGTACCCTGAGG
ATCTGGAGGTCACAGTTAAATGACACGGGGAACTACACAGTGAGGGTGGACACCATCAATGACACCCAGAGAGCAACT
GGCTGGCTCGAGATTCTAGAGTTGGAAATCCCGCAAATCTCGGTCAACACCACCTCCGTCGTAGATGGCGAGGATGCG
GTGGCTGCCACTTGCTACACCAATGACAGCCACGTCCAGTGGTATGTGAATCATGCACCGGTGTCCAGCAATTACCGG
ATGACCATCTCCCCGGACAACAAGACCCTCATCATCCGAATGTTCAGCCGCTTCGACTCACCACTTCAGTGCGGGATA
GAAATTCTCCCAGAGCTCATTCAGAAAAGTGACCTCGTCTATGTGACAGTGGCCTATGGGCCCTACAGTCTGCAGCTC
AGGAGCAGTCCCACTGACTTCAGTGGCATCCTGTCTGCTGAGATTTGCTCCCAGGTGGAGATGGAGTGCATCTCCTAT
TCCAGACCAGAATCCAAGTACCGCTGGATCCACAATGGCTCCCTCCTGAGCTTCTCAGAGAAGAACATCACCCTCCCA
AGTCTGACCTGGGACCAGATGGGCAGATACAGGTGCATCGCAGAGAACTCCGCCACCCAGCTGACCTTGTACGATGAA
GTCCACATCCAGGCACTCTGGCGCCGGCCTGTTGTCAGCAGAAGTTTCACCATCTCAGGCTCCTTGTTGGTGTTTCTC
ATCATATTCACAGTTCTGGGCTTCACCCACTTCCTTATGGTCCTGATCCGTGCCCTGTTCAGACATTACTCTACCAGA
CCCGGTGGGATCATTAGAGAGGCTGCAAAGATGCCCCATCTCAGCATCGCCAGTGACAGAGAGTTGGAACCACAGCGC
ATGTCGGATGAATGTCATGGTTAG

>CEACAM19 [Bos taurus]

ATGGAGATTCCTGCTGGGTCCCAGCACGCCTTCCCCAAGAGCCTCCTGCTCTCAGCCTCCATCCTGGCCCTCTGGGTC
CCCCAAGGCTCCTGGGCAGCCCTGCGCATCCAGAAGATTCCGGAGCAGCCTCAAATGAACCAGGACCTTCTCCTGTCT
GTCCAGGGCATCCCAAACACCTTCCAGGACTTCAGCTGGTACCTGGGGGAGGAGGCCAATGGCGGTACAATGTTATTC
ACCTACATCCCCAAGCTACTACGCCCCCAGAGGGATGGCAGTGCCATGCATCAGCGAGACATTGTTGGCTTCTCCAAT
GGCTCCATGCTGCTACGTCACGCCCAGCCCAGCGACAGCGGCACCTATCAGGTAGCTGTCACCATCAACCCTTCCTGG
ACCATGCGGGCCAAGACTGAGGTCCAGGTGGTCGAAAAGCCTAAGGAGCTGCCCGTTACCAACCCGCCTGTGAGTGCT
GGGATTGTGGCTGCTATCGTCATTGGATCTCTTGCCACTGGGTGTATGTCCGTCGGCATCATTGCCTATCTCCTGGTG
ACAAGAGGCTGGAGGGCTCAGAACCACAGGATAACAGCCACAGAGAAACCAGAGCTGGGCCCCAGTCATCGTGCTGGT
GATGACAGCATCTATGAAGCAATACCATCGCCAGTCCTCCTGGTGTCCCCTTGTGACACGGGGCCCATGAACACTGCC
ACACCCCCACCCCCACCCCCACCGCCCCCGGTGCCGCAGCCTGAGAACCACCCCTACCAGGATCTGCTGAACCCCGAC
CCTGCCCCGTACTGCCAGCTGGTGCCCACGCCCTGA

>CEACAM20 [Bos taurus]

ATGGAGTCCCCTGATCTACGGGGCCACCGCTGGGCAGGAATCCTGCTCTCAGCCTCACTTTTGACCGTGTGGAGCTTG
ACAGCTGCAGCCCAGATCTCCCGTGATGCTGTCATCCAAAGTGAGGAGGATATTGTTCCGTCTACATTTGGGGCCCCT
TGGTTACCCCAGACTCATGAATCTCTGGCCAAGCCCACCATTTCAGTCAGCCAGGGCACAGTCATAGAGCACAGGGAA

AATGTGACCTTCTACTGTGACACCCCAGACGTCAACATCACCATCCACTGGGTCTTCAACCACCAGCCCCTGCCATCC
CATGAGCGCATACAGCTGTCCACAGATGGCAAGACCCTCACCATCCTCAGTGTCCAGCGGAAGGATGCTGGGACGTAC
CAATGTGAAGCTTGGGGTGACCTCCAGATGAAGAGCAGTGACCCTACCTACCTGATTGTGTACTATGGCCCTGACTCA
GTCACGATCAAGGTGGAGCCTGGTGTACCCAACGGGGACACAGTTGAGGTGATGGAGGGCTCCAGTGTGACCTTCTCA
GCAGAAACTGAGTCTTACCCACAAGCTTCATATTCATGGTTTTTCTCCAATGACTCCAAGCCCATCACTTGGAGCTTG
TTCTTCAACATGAGCAATGTTACCATCCCTGCCGTGTCCAAGGAACACGAGGGCACCTACGCGTGCTTGGTGTCCAAT
GCTGCCGCCCAGAAGTCCCTCAGGGATGCTGTCAAAGTCCGCGTCCTTGAAAGAGTGACCAAGCCTTACGTCATGGCC
CCAAACCAGACTCTCGTGGAGGACACCAGCTCTGTGGTCCTGACCTGCCAGACCACCCATGAGGGAGTTGGAGTCCGG
TGGTTCCTGGGGGACCAGCTCCTCCAGTCCAGCGAGCACCTGGCACCCAACAACAGGGACCTGATCATCCACGGCCTT
CGGCGGAATGACACAGGGCCCTATGCGTGCGAGGTCTGGAACTGGGGCAGCCAGGCACGGAGTGAACCCCTGAAGTTG
AACATCAGCTACGGCCCCGATCGAATGTATTTCACCAGCGGGTCGGAGACCCTGGCGGACACCACGGTCAGTGTGGAG
CTCAACTCCCGCCTAACCCTGCGGTGTTGGGCTGAATCCCAGCCGGATGCTGAGTTTAACTGGACCCATGACAACACC
AGTGTGTACAAGGGGCAGCAGCTGGTTATCGAGGCCCTGACTTGGAAACACCAAGGGAATTACAGCTGCACAGCTTCT
AACTCTGTCACAATGCTGACCTGCTCTGCCTCGGTCATGGTCAGCGTCATAGGTCACCGGTCATCCCTCTCTGTAGGG
GCCATCGTTGGCATTACCGTCGGGATCCTGGCTATCGTTGCCCTGGCCATAGGGCTGGGCTGCTTCATACACACTGGA
AATGCCGAAGGGCTCTCAAGGAGAACAACAGAGCATACCGCCTATGAGAACATGACACCCACTTCTGAAGAGGGTCAC
CCTGAAGAGCTCGGTCGCAGCTGGCCCATGCCTGTGTATGCCAATGCACCCGCCATTGAAGGACAGATACCAGTCAAA
AAGATGCTGCCAGTAGACCCTCCAGAGCAATTATATGAGCAGTTATCACCTCCCACCAGCCACAGCCGGTACTCTCAT
GGCCCCAGGAAGCCATCCTCCAATCCCTTGGTCCCAACTCCACAAAAAGAAAATGCAGAGTCAAACTATGAGGCGCTT
GTGAATCCAGAACACAGCATCTACTGCCAAATCAACCGTTCCACCTAA
